# Entropy of Volatility Changes: Novel Method for Assessment of Regularity in Volatility Time Series

**DOI:** 10.3390/e27030318

**Published:** 2025-03-19

**Authors:** Joanna Olbryś

**Affiliations:** Faculty of Computer Science, Bialystok University of Technology, Wiejska 45a, 15-351 Białystok, Poland; j.olbrys@pb.edu.pl

**Keywords:** entropy, volatility, range-based daily data, symbolic time series analysis (STSA), stock market, information content, complexity, regularity

## Abstract

The goal of this research is to introduce and thoroughly investigate a new methodology for the assessment of sequential regularity in volatility time series. Three volatility estimators based on daily range data are analyzed: (1) the Parkinson estimator, (2) the Garman–Klass estimator, and (3) the Rogers–Satchell estimator. To measure the level of complexity of time series, the modified Shannon entropy based on symbol-sequence histograms is utilized. Discretization of the time series of volatility changes into a sequence of symbols is performed using a novel encoding procedure with two thresholds. Five main stock market indexes are analyzed. The whole sample covers the period from January 2017 to December 2023 (seven years). To check the robustness of our empirical findings, two sub-samples of equal length are investigated: (1) the pre-COVID-19 period from January 2017 to February 2020 and (2) the COVID-19 pandemic period from March 2020 to April 2023. An additional formal statistical analysis of the symbol-sequence histograms is conducted. The empirical results for all volatility estimators and stock market indexes are homogeneous and confirm that the level of regularity (in terms of sequential patterns) in the time series of daily volatility changes is high, independently of the choice of sample period. These results are important for academics and practitioners since the existence of regularity in the time series of volatility changes implies the possibility of volatility prediction.

## 1. Introduction

Modeling and forecasting stock market volatility has been the subject of vast empirical and theoretical investigation by academics and practitioners alike [1]. Undoubtedly, volatility has many financial applications, but a special feature of asset volatility is that it is not directly observable [2].

Nelson [3] emphasized that volatility is subject to sudden shifts, and the literature on this topic has uncovered several phenomena associated with market volatility changes. Positive serial correlations in volatility (known as volatility clustering), leverage effects, and leptokurtosis are among them [1]. For instance, volatility clustering means the tendency for volatility in financial markets to appear in bunches. In other words, large volatility changes tend to be followed by large changes, and small changes tend to be followed by small changes.

As Brooks [1] pointed out, financial markets are extremely complex, involving a very large number of different participants, each with different objectives and different sets of information. The consequence of this is that economic and financial data are usually far noisier and ‘more random’ than data from other disciplines. This evidence also concerns stock market volatility.

Taking the above into consideration, the complexity and the internal structure of the time series of volatility changes is a well-founded and important research issue. In other words, it is interesting to look at the properties and structure of volatility change time series.

According to the literature, entropy is a universal measure of system complexity. Specifically, entropy is a widely used indicator that summarizes the information content of a probability distribution, and Shannon information entropy [4] quantifies the expected value of information contained in a discrete distribution. As Shannon [4] emphasized, the proper symbolic encoding of information is a foundation of the mathematical theory of communication. Data analysis tools referred to as *symbolic time series analysis (STSA)* tools enable researchers to transform raw time series data into a series of discretized symbols that are processed to extract information. Unfortunately, there is no universal rule for locating an optimal partition for real data [5]; hence, several encoding procedures have been introduced for various applications [6,7,8,9,10,11,12,13,14]. However, it is worth emphasizing that all of these procedures are complexity reduction methods that differ in terms of their effectiveness, and they help to recognize and determine sequential regularity in data series [10].

In this context, the contribution of this research is twofold. First, a new methodology for the assessment of sequential regularity in volatility time series is introduced and thoroughly investigated. To measure the level of complexity of volatility time series, modified the Shannon entropy based on symbol-sequence histograms is utilized. Discretization of the time series of volatility changes into a sequence of symbols is performed using a novel encoding procedure with two thresholds. Three volatility estimators based on daily range data are utilized: (1) the Parkinson estimator, (2) the Garman–Klass estimator, and (3) the Rogers–Satchell estimator. Five main stock market indexes are analyzed. The whole sample covers a long period from January 2017 to December 2023 (seven years).

Second, two sub-samples of equal length are investigated to check the robustness of our empirical findings. These sub-samples are (1) the pre-COVID-19 period from January 2017 to February 2020 (38 months) and (2) the COVID-19 pandemic period from March 2020 to April 2023 (38 months). It is important to note that the empirical results for all volatility estimators and stock market indexes are homogeneous and unambiguously confirm that the level of regularity (in terms of sequential patterns) in the time series of daily volatility changes is high, independently of the choice of sample period.

The obtained results are entirely novel and have not been presented in the literature thus far. The findings of this study are important for academics and practitioners since the existence of regularity in the time series of volatility changes implies the possibility of volatility prediction.

The rest of this paper is organized in the following way. Section 2 presents the methodological background concerning volatility indicators and introduces a novel method for the estimation of the modified Shannon entropy based on the symbolic encoding of volatility change time series. Section 3 describes the results and presents a discussion of the experimental studies. The Section 4 summarizes the main findings and indicates some further research directions. The paper is supplemented with one Appendix A that contains additional comparative results of the symbol-sequence statistics.

## 2. Methodological Background

This section presents the methodological background concerning three range-based volatility indicators and introduces a novel method for the estimation of the modified Shannon entropy based on symbolic encoding of the time series of volatility changes.

### 2.1. Volatility Estimators Based on Daily Data

The literature documents that the volatility of the underlying financial instrument is a fundamental time-varying indicator, but, in fact, it is not directly observable. However, there are two major and well-known approaches to volatility estimation. The first approach is grounded in historical information on the underlying financial instrument, and it comprises the so-called realized volatility measures. The second approach extracts information from derivatives traded in financial markets, and the resulting volatility indicator is called an implied volatility measure [15].

In this study, volatility estimation from historical range data is employed. Range data are available for most financial instruments and intuitively have more information than return data for calculating volatility. For comparison, the standard deviation, as a classical and simple volatility estimator, is usually based on close-to-close prices only.

Among others, Yang and Zhang [16] emphasize that, according to the literature, more sophisticated indicators use additional information such as high, low, opening, and closing prices to achieve better accuracy in volatility estimation. Garman and Klass [17] point out that alternative effective estimators of volatility may be constructed from the historical opening, closing, high, and low prices. Parkinson [18] shows that the use of extreme daily values, i.e., the highest and lowest prices, provides a far superior volatility estimate. Rogers and Satchell [19] propose a method for variance estimation from high, low, and closing prices.

To incorporate opening, closing, high, and low prices, the respective logarithmic rates of returns on day *t* are given by the following three formulas:

- Open–close returns:
  (1) $$c_{t}=ln\left(C_{t}\right)-ln\left(O_{t}\right)=ln\left(\frac{C_{t}}{O_{t}}\right),$$
- Open–high returns:
  (2) $$h_{t}=ln\left(H_{t}\right)-ln\left(O_{t}\right)=ln\left(\frac{H_{t}}{O_{t}}\right),$$
- Open–low returns:
  (3) $$l_{t}=ln\left(L_{t}\right)-ln\left(O_{t}\right)=ln\left(\frac{L_{t}}{O_{t}}\right),$$

where $H_{t}$, $L_{t}$, $O_{t}$ and $C_{t}$ are the high, low, opening, and closing prices on day *t*, respectively.

In light of the existing literature, e.g., [20,21,22,23], three range-based volatility estimators, based on returns defined by Equations (1)–(3), are especially useful in empirical studies concerning financial markets. These estimators are the Parkinson estimator [18] (Definition 1), the Garman–Klass estimator [17] (Definition 2), and the Rogers–Satchell estimator [19] (Definition 3).

For consistency of presentation, all volatility estimators given by Definitions 1–3 are expressed as standard deviations and denoted by $v_{t}$ with a corresponding upper index.

**Definition** **1.** *The Parkinson [18] high–low range volatility estimator is defined as follows:*

(4) $$v_{t}^{P}=\sqrt{\frac{{\left(h_{t}-l_{t}\right)}^{2}}{4ln2}}=\sqrt{\frac{{\left[ln\left(\frac{H_{t}}{L_{t}}\right)\right]}^{2}}{4ln2}}$$

**Definition** **2.** *The Garman–Klass [17] range-based volatility estimator is defined by the following equation:*

(5) $$v_{t}^{GK}=\sqrt{0.5{\left(h_{t}-l_{t}\right)}^{2}-\left(2ln2-1\right)c_{t}^{2}}=\sqrt{0.5{\left[ln\left(\frac{H_{t}}{L_{t}}\right)\right]}^{2}-\left(2ln2-1\right)\cdot {\left[ln\left(\frac{C_{t}}{O_{t}}\right)\right]}^{2}}$$

**Definition** **3.** *The Rogers–Satchell [19] range-based volatility estimator is defined as follows:*

(6) $$v_{t}^{RS}=\sqrt{h_{t}\left(h_{t}-c_{t}\right)+l_{t}\left(l_{t}-c_{t}\right)}=\sqrt{ln\left(\frac{H_{t}}{O_{t}}\right)\cdot ln\left(\frac{H_{t}}{C_{t}}\right)+ln\left(\frac{L_{t}}{O_{t}}\right)\cdot ln\left(\frac{L_{t}}{C_{t}}\right)}$$

In light of the existing literature, there are pros and cons of range-based volatility estimators, but the main conclusions presented by various researchers are rather diverse. For instance, Parkinson [18] and Garman and Klass [17] prove that their estimators are much more efficient than the classical volatility estimator. In theory, the range volatility estimators proposed by Parkinson [18] and Garman and Klass [17] require geometric Brownian motion with zero drift, while the Rogers–Satchell estimator [19] allows a non-zero drift. Molnár [20] presents an overview of range-based volatility estimators. He emphasizes that a simple volatility estimator has, by definition, an efficiency of 1, while the Parkinson estimator [18] has an efficiency of 4.9, the Garman–Klass estimator [17] has an efficiency of 7.4, and the Rogers–Satchell estimator [19] has an efficiency of 6 for zero drift and larger than 2 for any drift. Moreover, Molnár [20] finds the Garman–Klass estimator to be the best volatility estimator based on daily data. Wiggins [24] argues that the extreme-value estimators of Parkinson [18] and Garman and Klass [17] are much more efficient than the close-to-close estimator. This observation is in accordance with intuition since range-based estimators incorporate the range of dispersion of prices over the entire day, not just the prices at the end of two consecutive days.

On the other hand, there are some disadvantages of range-based volatility estimators. For instance, the Garman–Klass estimator (Definition 2) utilizes the opening price, which is sensitive to effects associated with low stock liquidity at the beginning of quotations [25]. Moreover, the Rogers–Satchell estimator can take zero despite big price changes within a day [25]. This could happen when the opening price is equal to the high price and the closing price is equal to the low price or vice versa, i.e., the opening price is equal to the low price and the closing price is equal the high price (see Definition 3). However, the aforementioned problems concern stock prices rather than values of the stock market index.

### 2.2. Symbolic Encoding of Volatility Time Series

In this study, the financial time series of stock market indexes are investigated, and daily volatility changes are calculated as follows:

(7) $$\Delta v_{t}=v_{t}-v_{t-1},$$

where $v_{t}$ is a value of a particular volatility estimator on day *t*.

Three different time series of volatility changes are taken into consideration for each stock market index:

1. $\left\{\Delta v_{t}\right\}=\left\{\Delta v_{t}^{P}\right\}$, where $v_{t}^{P}$ is the volatility estimator given by Definition 1.
2. $\left\{\Delta v_{t}\right\}=\left\{\Delta v_{t}^{GK}\right\}$, where $v_{t}^{GK}$ is the volatility estimator given by Definition 2,
3. $\left\{\Delta v_{t}\right\}=\left\{\Delta v_{t}^{RS}\right\}$, where $v_{t}^{RS}$ is the volatility estimator given by Definition 3.

Schittenkopf et al. [15] emphasize that volatility changes could be modeled directly using symbolic encoding procedures. The authors propose the discretization process based on the binary STSA method with one threshold equal to zero.

According to the literature, different coding alphabets corresponding to different levels of discretization could be utilized to code the same raw data. A finite set *A* of possible *n* symbols is called an alphabet. Each subset of a sequence of *k* symbols is called a word [4,5,7,26].

Among others, Daw et al. [7] point out that binary encoding (A={0,1}, n=2) is suitable in many cases; however, higher values of n>2 enable the researcher to increase the discrimination of measurement details. Moreover, in the paper [10], six different STSA methods are investigated and compared, and the empirical findings confirm that the methods with two thresholds (ternary encoding) work better than the methods with one threshold (binary encoding).

Therefore, in this research, discretization of the series of volatility changes $\left\{\Delta v_{t}\right\}$ into sequences of symbols $\left\{s_{t}\right\}$ is performed using a novel encoding procedure with two thresholds. The finite alphabet A={0,1,2}, n=3 is used. The proposed new STSA method is defined as follows:

**Definition** **4.** *A sequence $\left\{s_{t}\right\}$ of symbols is defined according to*

(8) $$s_{t}=\left\{\begin{array}{cc} 0 & if\;\Delta v_{t}\leq \left(\bar{\Delta v}-\sigma \right)\\ 1 & if\;\left(\bar{\Delta v}-\sigma \right)<\Delta v_{t}\leq \left(\bar{\Delta v}+\sigma \right)\\ 2 & if\;\Delta v_{t}>\left(\bar{\Delta v}+\sigma \right) \end{array}\right.$$

*where $\bar{\Delta v}$ is the mean of changes in volatility, σ is the standard deviation of changes in volatility, and $\left(\bar{\Delta v}-\sigma \right)$ and $\left(\bar{\Delta v}+\sigma \right)$ are the thresholds of the time series of volatility changes $\left\{\Delta v_{t}\right\}$.*

### 2.3. Entropy of Volatility Changes

As mentioned in the Introduction, Shannon information entropy [4] quantifies the expected value of information contained in a discrete distribution. The Shannon entropy of *k*-th order (Definition 5) is an information-theoretic measure for symbol-sequence frequencies [10].

**Definition** **5.** *The Shannon entropy of k-th order, H(k), is defined according to the following:*

(9) $$H\left(k\right)=-\sum_{i}p_{i}\cdot log_{2}\left(p_{i}\right)$$

*where $p_{i}$ is the probability of finding the i-th sequence of length k.*

The probability $p_{i}$ is approximated by the number of times the *i*-th sequence is found in the original symbolic string divided by the number of all non-zero sequences of length *k*. In this research, following the paper [5], we utilize the definition of the modified Shannon entropy $H_{s}\left(k\right)$, which is just a normalized form of the Shannon entropy H(k).

**Definition** **6.** *The modified (normalized) Shannon entropy $0\leq H_{s}\left(k\right)\leq 1$ based on the symbolic representation of time series is defined according to the following:*

(10) $$H_{s}\left(k\right)=\frac{-1}{log_{2}N}\cdot \sum_{i}p_{i}\cdot log_{2}\left(p_{i}\right)$$

*where N is the total number of observed sequences of length k with non-zero frequency, i is the index of a sequence, and $p_{i}$ is the probability of finding the i-th sequence of length k. It is assumed that $0\cdot log_{2}0=0$.*

## 3. Experimental Studies: Results and Discussion

This section describes the real database and presents our empirical findings regarding this novel methodology for the assessment of the information content of volatility time series obtained using the three volatility estimators defined in the previous section (Definitions 1–3).

### 3.1. Data Description

The data set includes daily observations for the major five stock market indices (S&P500, CAC40, FTSE100, DAX, and Nikkei225). The whole sample covers the period from January 2017 to December 2023 (seven years).

Moreover, two sub-samples of equal length are investigated: the pre-COVID-19 period and the COVID-19 pandemic outbreak. To determine the COVID-19 pandemic period, we use the World Health Organization (WHO) information [27]. It is assumed that this period started in March 2020 since on 11 March 2020, the WHO officially declared the COVID-19 outbreak to be a global pandemic. By analogy, the COVID-19 period ended on 5 May 2023 based on the WHO declaration [28].

It is obvious that the numbers of trading days within the analyzed periods in various countries are not equal since they are subject to different national and religious holidays, unexpected events, and so on.

Therefore, for simplicity and in order to ensure the comparability of our empirical findings, both sub-periods are measured in full months:

1. The pre-COVID-19 pandemic period from January 2017 to February 2020 (38 months);
2. The COVID-19 pandemic period from March 2020 to April 2023 (38 months).

Table 1, Table 2 and Table 3 include brief overviews of the information about the analyzed indices and the summarized statistics for daily volatility changes (calculated based on Equation (7)) within the whole sample period and two investigated sub-periods, for three volatility estimators given by Definitions 1–3. The indices are labeled with ticker symbols. One can observe that the basic statistics for volatility changes are very similar for all investigated equity markets, within all analyzed periods. Specifically, the mean values of volatility changes are almost equal to zero and negative within the whole sample in the COVID-19 period, while within the pre-COVID-19 period, they are almost equal to zero but positive. Moreover, in general, the standard deviation values are slightly higher within the COVID-19 period, which is in accordance with our expectations. To summarize, the results obtained for three volatility estimators are consistent in the case of all analyzed stock market indexes.

### 3.2. Discretization of Time Series of Volatility Changes

As pointed out in Section 2.2, in this research, discretization of the series of volatility changes $\left\{\Delta v_{t}\right\}$ into sequences of symbols $\left\{s_{t}\right\}$ is performed using a novel encoding procedure with two thresholds, given by Definition 4.

Direct identification of symbolic dynamic patterns in a particular time series of volatility changes consists of two steps. The first step encompasses symbolic encoding with thresholds based on Definition 4. The second step is the construction of symbol sequences. Each possible sequence is represented in terms of a unique code given by a natural number [10]. Table 4 contains the proposed assigned codes of all possible sequences in the case of the alphabet A={0,1,2} (n=3) and the k=3 length of a code sequence ($n^{k}=3^{3}=27$ natural numbers). However, the coding natural numbers and the order of sequences could be different and chosen arbitrarily, e.g., [10]. In this research, the most frequently observed sequence (1,1,1) is the first, which determines the symbol-sequence histogram charts.

### 3.3. Symbol-Sequence Histograms of Encoded Series of Volatility Changes

The dynamics of a structure of patterns in encoded real data can be described by a *k*-histogram that presents frequencies of possible symbol sequences. The empirical distribution visualized by such a histogram allows for the comparison and deep investigation of symbol sequences. According to the literature, direct presentation of the frequencies with histograms provides a convenient way of observing possible patterns in time series [5,7,10,14,29]. An important issue in pattern recognition is finding similarities between two histograms. In the literature, a number of measures for computing the distance between two histograms have been proposed and used in various applications [30,31].

A formal comparison of symbol-sequence histograms can be conducted using classical distance statistics (see e.g., [31], p. 1360). In this study, two distance metrics are utilized: (1) the Euclidean norm and (2) the Manhattan norm.

The Euclidean norm is given by Equation (11):

(11) $$E_{XY}\left(k\right)=\sqrt{\sum_{i}{\left(X_{i}-Y_{i}\right)}^{2}},$$

where $X_{i}$ and $Y_{i}$ are the empirical frequencies of individual sequences for the sequence codes *i* given the *k*-histograms *X* and *Y*.

The Manhattan norm is given by the following Equation (12):

(12) $$M_{XY}\left(k\right)=\sum_{i}\left|X_{i}-Y_{i}\right|,$$

where the notation is as in Equation (11).

Both aforementioned norms work like metrics in the space of all possible symbol sequences, providing measures of the distance between different *k*-histograms. In general, greater distances indicate that the dynamics in the data is increasingly different. Both metrics are especially useful in the case of two samples with an equal length [14].

### 3.4. Empirical Results Within the Whole Sample Period: The Modified Shannon Entropy of Volatility Changes, Histograms, and Symbol-Sequence Statistics

This section presents and discusses our empirical findings within the whole sample period from January 2017 to December 2023. All calculations were conducted with a dedicated program that was created using the Jupiter Notebook—an interactive computing platform based on the Python 3.13.2 language.

Table 5 provides the summarized findings of the modified Shannon entropy of volatility changes based on the novel encoding procedure (Definition 4) for three volatility estimators and five investigated stock markets. As one can observe, all results are homogeneous. The level of entropy is very similar for all cases, but the maximum of the entropy value is observed for the DAX index (Germany).

**Figure 1:**
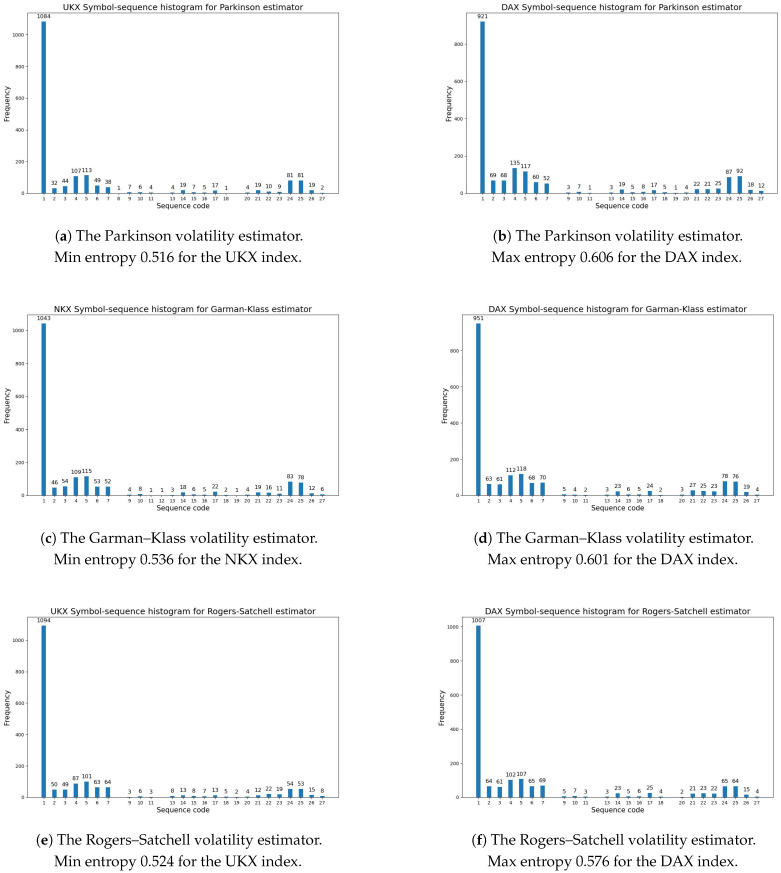
The symbol-sequence histograms for the max and min values of the modified Shannon entropy given by Definition 6 within the whole sample period from January 2017 to December 2023 (based on Table 5): (**a**) The Parkinson volatility estimator (Definition 1), the UKX index. (**b**) The Parkinson volatility estimator (Definition 1), the DAX index. (**c**) The Garman–Klass volatility estimator (Definition 2), the NKX index. (**d**) The Garman–Klass volatility estimator (Definition 2), the DAX index. (**e**) The Rogers–Satchell volatility estimator (Definition 3), the UKX index. (**f**) The Rogers–Satchell volatility estimator (Definition 3), the DAX index.

Figure 1 presents the symbol-sequence histograms for the max and min values of the modified Shannon entropy given by Definition 6 within the whole sample period (based on Table 5).

Table 6 reports the codes of sequences with zero frequency (based on symbol-sequence histograms). These codes are defined in Table 4. To summarize, the results reveal that for almost all cases, sequence No. 8 (0,0,0) and No. 12 (2,2,2) did not appear. Based on Definition 4, sequence No. 8 (0,0,0) means that three consecutive daily changes in volatility were extremely low (i.e., lower than the first threshold), while sequence No. 12 (2,2,2) means that three consecutive daily changes in volatility were extremely high (i.e., higher than the second threshold).

Table 7 documents the five most frequently observed sequences within the whole sample period (based on the symbol-sequence histograms). The codes of sequences are defined in Table 4. The results are homogeneous for all investigated volatility estimators and all analyzed stock markets. Sequence No. 1 (1,1,1,) dominated decidedly (which is easily visible in Figure 1). This sequence means that three successive daily changes in volatility were not extremely high or low, but lay between thresholds. Moreover, one can observe that sequence No. 4 (0,1,1) and No. 5 (1,1,2) repeated in all cases. The evidence is that the level of regularity in daily volatility changes was high for all volatility estimators.

### 3.5. The Comparative Empirical Findings for the Pre-COVID-19 and COVID-19 Periods: The Modified Shannon Entropy of Volatility Changes, Histograms, and Statistics

In this subsection, the comparative empirical results within the pre-COVID-19 and COVID-19 periods for all investigated stock market indexes are discussed to check the robustness of our empirical findings.

#### 3.5.1. The Comparative Empirical Findings for the Parkinson Volatility Estimator

Table 8 contains the results for the modified Shannon entropy of volatility changes for the Parkinson volatility estimator. The column entitled ‘Change in Entropy’ documents changes in entropy between the periods before and during the pandemic. The up arrows show an entropy increase, while the down arrows illustrate an entropy decrease. The results are rather diverse since the modified Shannon entropy decreased for three indexes (i.e., CAC, UKX, and DAX), while entropy increased for two indexes (i.e., SPX and NKX).

**Figure 2:**
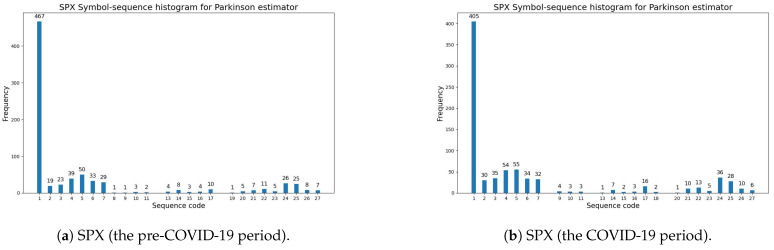

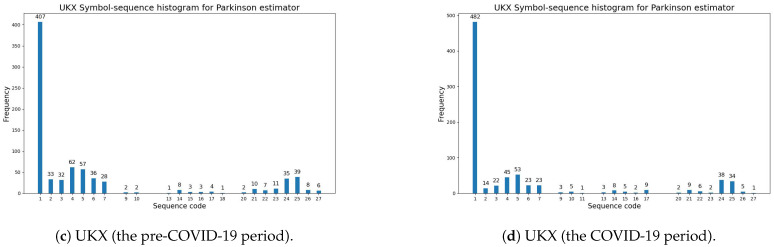
The Parkinson volatility estimator (Definition 1). A comparative analysis of symbol-sequence histograms in the case of positive (SPX) and negative (UKX) changes in the modified Shannon entropy, given by Definition 6, within the pre-COVID-19 and COVID-19 periods (based on Table 8): (**a**) SPX (USA)—the pre-COVID-19 period; (**b**) SPX (USA)—the COVID-19 period; (**c**) UKX (U.K.)—the pre-COVID-19 period; (**d**) UKX (U.K.)—the COVID-19 period.

Figure 2 exemplifies the symbol-sequence histograms for one case of a positive change in the modified Shannon entropy (the SPX index) and for one case of a negative change in entropy (the UKX index), based on Table 8 (the Parkinson volatility estimator). The numbers of the most frequently observed sequences within the pre-COVID-19 and COVID-19 periods (which are visible in Figure 2) are reported in Table A3 and Table A4 (Appendix A). The numbers of the sequences with zero frequency within the pre-COVID-19 and COVID-19 periods are presented in Table A1 and Table A2 (Appendix A).

#### 3.5.2. Comparative Empirical Findings for the Garman–Klass Volatility Estimator

Table 9 reports the results for the modified Shannon entropy of volatility changes for the Garman–Klass volatility estimator. The column entitled ‘Change in Entropy’ documents changes in entropy before and during the pandemic period. The up arrows show an entropy increase, while the down arrows illustrate an entropy decrease. The results are more homogeneous than for the Parkinson estimator since the modified Shannon entropy decreased for four indexes (i.e., CAC, UKX, DAX, and NKX), while entropy increased for only one index (SPX).

**Figure 3:**
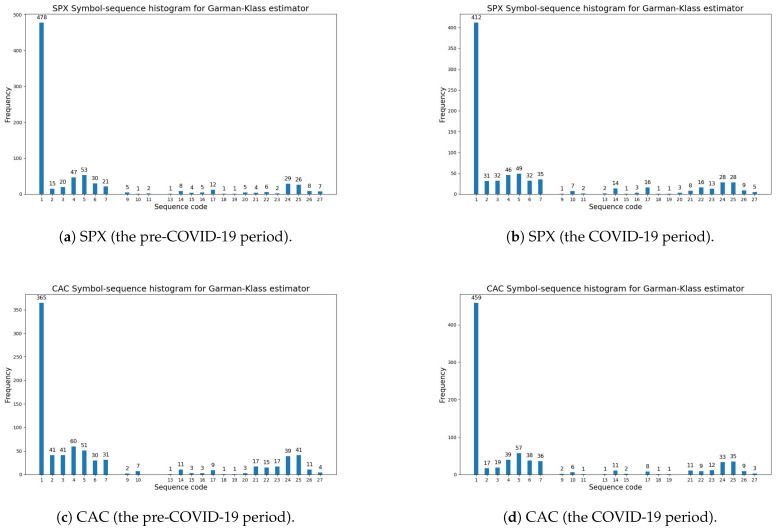
The Garman–Klass volatility estimator (Definition 2). A comparative analysis of symbol-sequence histograms in the case of positive (SPX) and negative (CAC) changes in the modified Shannon entropy, given by Definition 6, within the pre-COVID-19 and COVID-19 periods (based on Table 9): (**a**) SPX (USA)—the pre-COVID-19 period; (**b**) SPX (USA)—the COVID-19 period; (**c**) CAC (France)—the pre-COVID-19 period; (**d**) CAC (France)—the COVID-19 period.

Figure 3 illustrates the symbol-sequence histograms for one case of a positive change in entropy (the SPX index) and for one case of a negative change in entropy (the CAC index), based on Table 9 (the Garman–Klass volatility estimator). The numbers of the most frequently observed sequences within the pre-COVID-19 and COVID-19 periods (which are visible in Figure 3) are reported in Table A3 and Table A4 (Appendix A). The numbers of the sequences with zero frequency within the pre-COVID-19 and COVID-19 periods are presented in Table A1 and Table A2 (Appendix A).

#### 3.5.3. Comparative Empirical Findings for the Rogers–Satchell Volatility Estimator

This subsection reports the main empirical findings for the Rogers–Satchell volatility estimator.

Table 10 shows the results for the modified Shannon entropy of volatility changes for the Rogers–Satchell volatility estimator. The column entitled ‘Change in Entropy’ documents changes in entropy before and during the pandemic period. The up arrows show an entropy increase, while the down arrows illustrate an entropy decrease. Similarly to the Garman–Klass volatility estimator, the results are more homogeneous than for the Parkinson estimator because the modified Shannon entropy decreased for four indexes (i.e., CAC, UKX, DAX, and NKX), while entropy increased for only one index (SPX).

**Figure 4:**
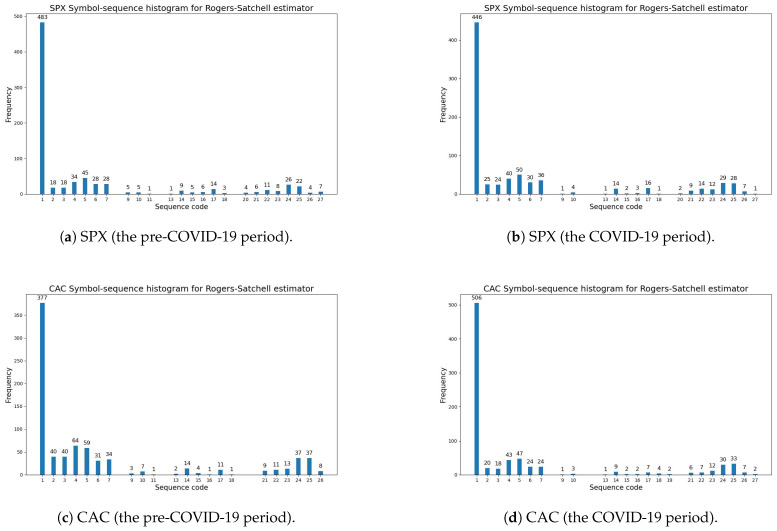
The Rogers–Satchell volatility estimator (Definition 3). A comparative analysis of symbol-sequence histograms in the case of positive (SPX) and negative (CAC) changes in the modified Shannon entropy, given by Definition 6, within the pre-COVID-19 and COVID-19 periods (based on Table 10): (**a**) SPX (USA)—the pre-COVID-19 period; (**b**) SPX (USA)—the COVID-19 period; (**c**) CAC (France)—the pre-COVID-19 period; (**d**) CAC (France)—the COVID-19 period.

Figure 4 visualizes the symbol-sequence histograms for one case of a positive change in entropy (the SPX index) and for one case of a negative change in entropy (the CAC index) based on Table 10 (the Rogers–Satchell volatility estimator). The numbers of the most frequently observed sequences within the pre-COVID-19 and COVID-19 periods (which are visible in Figure 4) are reported in Table A3 and Table A4 (Appendix A).

#### 3.5.4. Statistical Comparison of Symbol-Sequence Histograms

In this subsection, the statistical comparison of symbol-sequence histograms is discussed. As pointed out in Section 3.3, a formal comparison of such histograms can be conducted using classical distance statistics.

**Figure 5:**
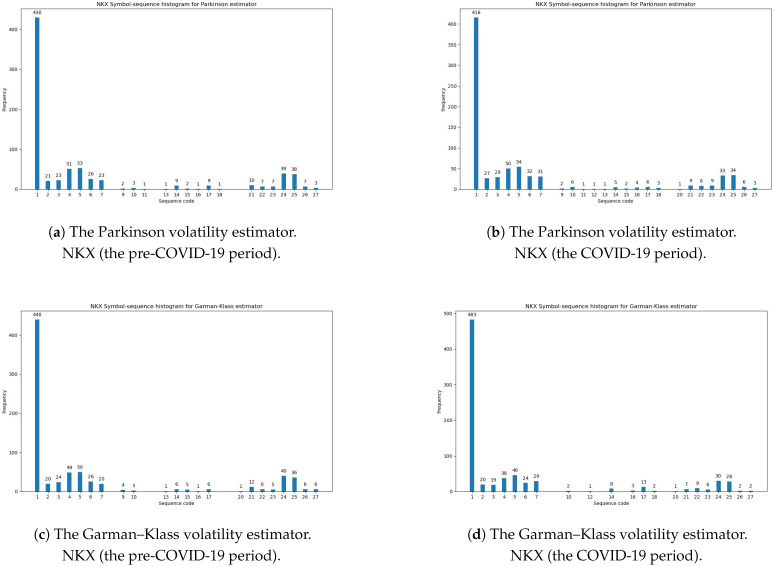

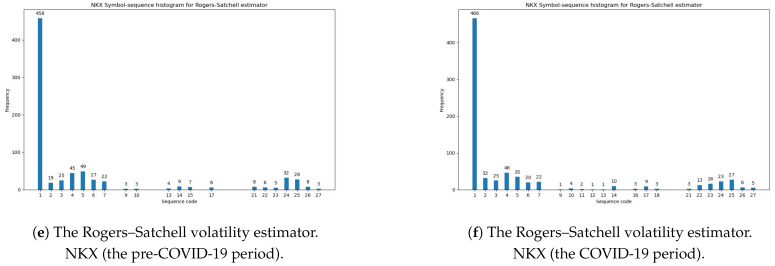
The symbol-sequence histograms for the NKX index: (**a**) The Parkinson volatility estimator (Definition 1), the pre-COVID-19 period. (**b**) The Parkinson volatility estimator (Definition 1), the COVID-19 period. (**c**) The Garman–Klass volatility estimator (Definition 2), the pre-COVID-19 period. (**d**) The Garman–Klass volatility estimator (Definition 2), the COVID-19 period. (**e**) The Rogers–Satchell volatility estimator (Definition 3), the pre-COVID-19 period. (**f**) The Rogers–Satchell volatility estimator (Definition 3), the COVID-19 period.

Table 11 documents the summarized statistical results of the differences between the two histograms for the three investigated volatility estimators during the pre-COVID-19 and COVID-19 periods. The smaller distance confirms that the dynamics in daily changes in volatility is smaller. In light of our results, the minimal values of the Euclidean and Manhattan norms for all volatility estimators were obtained for the NKX index (Japan). This evidence is directly observable in Table A3 and Table A4 (Appendix A) and in Figure 5. The analyses of the robustness of our empirical findings within the two sub-periods indicate that the level of regularity in volatility changes was especially stable for the Japanese stock market, regardless of the choice of sub-sample.

## 4. Discussion and Conclusions

The main methodological contribution of this study was the introduction and investigation of a novel method for the assessment of sequential regularity in volatility time series. To measure the level of complexity of these series, the modified Shannon entropy based on symbol-sequence histograms was employed. Discretization of a time series of volatility changes into a sequence of symbols was performed using the new encoding procedure. Three volatility estimators based on daily range data were analyzed: (1) the Parkinson estimator, (2) the Garman–Klass estimator, and (3) the Rogers–Satchell estimator. The goal was to explore the internal structure of the time series of volatility changes.

The main empirical contribution of this study was the examination of the new procedure within the whole sample period and within two sub-samples of equal length to check the robustness of our empirical findings. To summarize, the empirical results for all volatility estimators and stock market indexes were homogeneous and confirmed that the level of regularity in time series of daily volatility changes was high, independently of the choice of sample period.

The presented findings contribute to the broad debate about entropy-based applications in economics and finance. Specifically, our results contribute to the existing literature on the topic concerning the regularity and predictability of financial markets. In general, the previous literature documented that entropy of various financial asset return time series usually significantly falls during extreme-event periods such as financial crises, pandemic periods, wars, etc. (see, for instance, [10,12,32,33,34,35,36]). In other words, the regularity and predictability of asset returns usually rise due to the more frequent existence of patterns.

Although the literature documented that extreme events are possible determinants of volatility changes [3], our results indicated that the time series of daily volatility changes were rather stable and quite regular for all range-based volatility estimators and independently of the sample period choice. In general, these empirical results are unambiguous despite the fact that for the Garman–Klass and Rogers–Satchell volatility estimators, the results were slightly more homogeneous than for the Parkinson estimator. The most probable reason for this phenomenon is that the Parkinson estimator incorporates high and low prices only, while the Garman–Klass and Rogers–Satchell volatility estimators are based on opening, closing, high, and low prices (see Definitions 1–3). It seems that the latter two volatility estimators work slightly better.

The novel results of the presented research might be interesting both for academics and practitioners. The existence of regularity (in terms of sequential patterns) in time series of daily volatility changes implies the possibility of volatility prediction. There is an agreement in the literature that range-based volatility models incorporate additional information about quotations within a day, and therefore, such models outperform models based on closing prices only (see, e.g., [25,37] and the references therein). In other words, daily high, low, opening, and closing prices can improve the accuracy of volatility estimation, and therefore can also improve volatility forecasts, especially during various extreme-event periods [22]. Range-based volatility models that are formulated on the basis of price range data give more accurate forecasts of volatility than those based on the GARCH model [21].

Since the topic of entropy-based applications is crucial in finance, a promising avenue for further research could be an investigation of the complexity of other financial asset time series, e.g., Exchange-Traded Fund (ETF) time series, which are relatively novel and interesting investment opportunities [38]. However, literature concerning the topic of the information content of ETF time series is rare [39].

## Figures and Tables

**Table 1:** The Parkinson volatility estimator (Definition 1): basic statistics for daily volatility changes within the whole sample and two sub-samples.

Country	Index	The Whole Sample			Pre-COVID-19			COVID-19		
		N	Mean	Std. Dev.	N	Mean	Std. Dev.	N	Mean	Std. Dev.
USA	SPX	1759	$-2\times 10^{-7}$	0.003	793	$1\times 10^{-5}$	0.002	797	$-1\times 10^{-5}$	0.003
France	CAC	1791	$-1\times 10^{-6}$	0.003	806	$1\times 10^{-5}$	0.002	813	$-1\times 10^{-5}$	0.003
U.K.	UKX	1765	$-1\times 10^{-6}$	0.003	799	$2\times 10^{-5}$	0.002	797	$-2\times 10^{-5}$	0.004
Germany	DAX	1774	$-4\times 10^{-6}$	0.003	795	$3\times 10^{-6}$	0.003	807	$-5\times 10^{-6}$	0.004
Japan	NKX	1774	$-3\times 10^{-6}$	0.003	769	$1\times 10^{-5}$	0.003	775	$-1\times 10^{-5}$	0.003

N denotes the number of sample observations. The indexes are labeled with ticker symbols.

**Table 2:** The Garman–Klass volatility estimator (Definition 2): basic statistics for daily volatility changes within the whole sample and two sub-samples.

Country	Index	The Whole Sample			Pre-COVID-19			COVID-19		
		N	Mean	Std. Dev.	N	Mean	Std. Dev.	N	Mean	Std. Dev.
USA	SPX	1759	$-3\times 10^{-7}$	0.004	793	$2\times 10^{-5}$	0.003	797	$-2\times 10^{-5}$	0.005
France	CAC	1791	$-1\times 10^{-6}$	0.004	806	$2\times 10^{-5}$	0.003	813	$-1\times 10^{-5}$	0.005
U.K.	UKX	1765	$-2\times 10^{-6}$	0.004	799	$3\times 10^{-5}$	0.003	797	$-3\times 10^{-5}$	0.004
Germany	DAX	1774	$-4\times 10^{-6}$	0.004	795	$1\times 10^{-5}$	0.003	807	$-1\times 10^{-5}$	0.005
Japan	NKX	1774	$-3\times 10^{-6}$	0.004	769	$1\times 10^{-5}$	0.004	775	$-1\times 10^{-5}$	0.004

N denotes the number of sample observations. The indexes are labeled with ticker symbols.

**Table 3:** The Rogers–Satchell volatility estimator (Definition 3): basic statistics for daily volatility changes within the whole sample and two sub-samples.

Country	Index	The Whole Sample			Pre-COVID-19			COVID-19		
		N	Mean	Std. Dev.	N	Mean	Std. Dev.	N	Mean	Std. Dev.
USA	SPX	1759	$-1\times 10^{-7}$	0.005	793	$3\times 10^{-5}$	0.004	797	$-3\times 10^{-5}$	0.006
France	CAC	1791	$-1\times 10^{-6}$	0.005	806	$2\times 10^{-5}$	0.003	813	$-1\times 10^{-5}$	0.006
U.K.	UKX	1765	$-2\times 10^{-6}$	0.005	799	$3\times 10^{-5}$	0.003	797	$-3\times 10^{-5}$	0.006
Germany	DAX	1774	$-3\times 10^{-6}$	0.005	795	$1\times 10^{-5}$	0.004	807	$-5\times 10^{-6}$	0.006
Japan	NKX	1774	$-2\times 10^{-6}$	0.004	769	$2\times 10^{-5}$	0.004	775	$-1\times 10^{-5}$	0.005

N denotes the number of sample observations. The indexes are labeled with ticker symbols.

**Table 4:** The codes of all possible sequences (words) for the alphabet A={0,1,2} and k=3.

The Codes of Sequences for the Alphabet A={0,1,2} and k=3
$\begin{array}{ccccccccc} 1 & 2 & 3 & 4 & 5 & 6 & 7 & 8 & 9\\ \left(1,1,1\right) & \left(1,1,0\right) & \left(1,0,1\right) & \left(0,1,1\right) & \left(1,1,2\right) & \left(1,2,1\right) & \left(2,1,1\right) & \left(0,0,0\right) & \left(0,0,1\right)\\ 10 & 11 & 12 & 13 & 14 & 15 & 16 & 17 & 18\\ \left(0,1,0\right) & \left(1,0,0\right) & \left(2,2,2\right) & \left(0,0,2\right) & \left(0,2,0\right) & \left(2,0,0\right) & \left(2,2,0\right) & \left(2,0,2\right) & \left(0,2,2\right)\\ 19 & 20 & 21 & 22 & 23 & 24 & 25 & 26 & 27\\ \left(2,2,1\right) & \left(1,2,2\right) & \left(0,1,2\right) & \left(0,2,1\right) & \left(1,0,2\right) & \left(1,2,0\right) & \left(2,0,1\right) & \left(2,1,0\right) & \left(2,1,2\right) \end{array}$

**Table 5:** The modified Shannon entropy of volatility changes based on the novel STSA method (Definition 4) within the whole sample period from January 2017 to December 2023.

Country	Index	Parkinson Estimator (Definition 1)	Garman–Klass Estimator (Definition 2)	Rogers–Satchell Estimator (Definition 3)
USA	SPX	0.584	0.577	0.557
France	CAC	0.592	0.580	0.547
U.K.	UKX	0.516	0.571	0.524
Germany	DAX	0.606	0.601	0.576
Japan	NKX	0.560	0.536	0.540
Min	–	0.516	0.536	0.524
Max	–	0.606	0.601	0.576

The indexes are labeled with ticker symbols.

**Table 6:** The sequences with zero frequency within the whole sample period from January 2017 to December 2023.

	The Sequences with Zero Frequency Within the Whole Sample Period				
	SPX	CAC	UKX	DAX	NKX
Parkinson	No. 12 (2,2,2)	No. 8 (0,0,0)	No. 12 (2,2,2)	No. 8 (0,0,0)	No. 8 (0,0,0)
Estimator		No. 12 (2,2,2)	No. 19 (2,2,1)	No. 12 (2,2,2)	
Garman–Klass	No. 8 (0,0,0)	No. 8 (0,0,0)	No. 8 (0,0,0)	No. 8 (0,0,0)	No. 8 (0,0,0)
Estimator	No. 12 (2,2,2)	No. 12 (2,2,2)	No. 12 (2,2,2)	No. 12 (2,2,2)	
				No. 19 (2,2,1)	
Rogers–Satchell	No. 8 (0,0,0)	No. 8 (0,0,0)	No. 8 (0,0,0)	No. 8 (0,0,0)	No. 8 (0,0,0)
Estimator	No. 12 (2,2,2)	No. 11 (1,0,0)	No. 12 (2,2,2)	No. 12 (2,2,2)	
	No. 19 (2,2,1)			No. 19 (2,2,1)	

Based on symbol-sequence histograms. Notation as in Table 4.

**Table 7:** The five most frequently observed sequences within the whole sample period from January 2017 to December 2023.

	The Five Most Frequently Observed Sequences Within the Whole Sample Period				
	SPX	CAC	UKX	DAX	NKX
Parkinson	No. 1 (981 times)	No. 1 (971 times)	No. 1 (1084 times)	No. 1 (921 times)	No. 1 (994 times)
Estimator	No. 5 (104 times)	No. 4 (119 times)	No. 5 (113 times)	No. 4 (135 times)	No. 4 (117 times)
	No. 4 (90 times)	No. 5 (113 times)	No. 4 (107 times)	No. 5 (117 times)	No. 5 (117 times)
	No. 6 (73 times)	No. 25 (81 times)	No. 24 (81 times)	No. 25 (92 times)	No. 24 (88 times)
	No. 7 (69 times)	No. 24 (76 times)	No. 25 (81 times)	No. 24 (87 times)	No. 25 (86 times)
Garman–Klass	No. 1 (1015 times)	No. 1 (985 times)	No. 1 (998 times)	No. 1 (951 times)	No. 1 (1043 times)
Estimator	No. 5 (100 times)	No. 5 (115 times)	No. 5 (116 times)	No. 5 (118 times)	No. 5 (115 times)
	No. 4 (86 times)	No. 4 (109 times)	No. 4 (113 times)	No. 4 (112 times)	No. 4 (109 times)
	No. 7 (67 times)	No. 24 (78 times)	No. 24 (75 times)	No. 24 (78 times)	No. 24 (83 times)
	No. 6 (66 times)	No. 25 (78 times)	No. 3 (67 times)	No. 25 (76 times)	No. 25 (78 times)
Rogers–Satchell	No. 1 (1055 times)	No. 1 (1055 times)	No. 1 (1094 times)	No. 1 (1007 times)	No. 1 (1055 times)
Estimator	No. 5 (92 times)	No. 5 (107 times)	No. 5 (101 times)	No. 5 (107 times)	No. 4 (107 times)
	No. 4 (77 times)	No. 4 (105 times)	No. 4 (87 times)	No. 4 (102 times)	No. 5 (93 times)
	No. 7 (66 times)	No. 25 (69 times)	No. 7 (64 times)	No. 7 (69 times)	No. 24 (68 times)
	No. 6 (64 times)	No. 6 (65 times)	No. 6 (63 times)	No. 6 (65 times)	No. 25 (68 times)

Based on symbol-sequence histograms. Notation as in Table 4.

**Table 8:** The Parkinson volatility estimator (Definition 1): the modified Shannon entropy of volatility changes based on the novel STSA method (Definition 4).

The Parkinson Volatility Estimator: The Modified Shannon Entropy of Volatility Changes				
Country	Index	Pre-COVID-19	COVID-19	Change in Entropy
USA	SPX	0.557	0.628	0.071 ↑
France	CAC	0.637	0.583	−0.054 ↓
U.K.	UKX	0.622	0.538	−0.084 ↓
Germany	DAX	0.650	0.611	−0.039 ↓
Japan	NKX	0.578	0.591	0.013 ↑
–	Min	0.557	0.538	–
–	Max	0.650	0.628	–

The indexes are labeled with ticker symbols.

**Table 9:** The Garman–Klass volatility estimator (Definition 2): the modified Shannon entropy of volatility changes based on the novel STSA method (Definition 4).

The Garman–Klass Volatility Estimator: The Modified Shannon Entropy of Volatility Changes				
Country	Index	Pre-COVID-19	COVID-19	Change in Entropy
USA	SPX	0.536	0.624	0.088 ↑
France	CAC	0.677	0.579	−0.098 ↓
U.K.	UKX	0.674	0.601	−0.073 ↓
Germany	DAX	0.660	0.580	−0.080 ↓
Japan	NKX	0.576	0.533	−0.043 ↓
–	Min	0.536	0.533	–
–	Max	0.677	0.624	–

The indexes are labeled with ticker symbols.

**Table 10:** The Rogers–Satchell volatility estimator (Definition 3): the modified Shannon entropy of volatility changes based on the novel STSA method (Definition 4).

The Rogers–Satchell Volatility Estimator: The Modified Shannon Entropy of Volatility Changes				
Country	Index	Pre-COVID-19	COVID-19	Change in Entropy
USA	SPX	0.551	0.591	0.040 ↑
France	CAC	0.669	0.525	−0.144 ↓
U.K.	UKX	0.649	0.547	−0.102 ↓
Germany	DAX	0.644	0.565	−0.079 ↓
Japan	NKX	0.573	0.553	−0.020 ↓
–	Min	0.551	0.525	–
–	Max	0.669	0.591	–

The indexes are labeled with ticker symbols.

**Table 11:** The summarized statistical results of the differences between the two histograms for the three investigated volatility estimators within the pre-COVID-19 and COVID-19 periods.

Country	Index	The Euclidean Norm (Equation (11))			The Manhattan Norm (Equation (12))		
		Parkinson Estimator	Garman–Klass Estimator	Rogers–Satchell Estimator	Parkinson Estimator	Garman–Klass Estimator	Rogers–Satchell Estimator
USA	SPX	67.65	73.09	42.19	154	174	122
France	CAC	54.55	103.24	135.78	144	222	268
U.K.	UKX	82.35	68.98	89.22	186	164	198
Germany	DAX	47.33	95.44	103.11	128	190	222
Japan	NKX	21.86	49.32	29.33	74	134	106
	Min	21.86	49.32	29.33	74	134	106
	Max	82.35	103.24	135.78	186	222	268

Based on symbol-sequence histograms. The indexes are labeled with ticker symbols.

## Data Availability

The data were obtained free of charge from the Stooq website (https://stooq.pl, (accessed on 30 June 2024)).

## Appendix A. Symbol-Sequence Statistics: Additional Comparative Results

Table A1 and Table A2 report the sequences with zero frequency within the pre-COVID-19 and COVID-19 periods. The main evidence is that these sequences are almost the same for all investigated volatility estimators and stock market indexes.

**Table A1:** The sequences with zero frequency within the pre-COVID-19 period.

	The Sequences with Zero Frequency Within the Pre-COVID-19 Period				
	SPX	CAC	UKX	DAX	NKX
Parkinson	No. 12, No. 18	No. 8, No. 11,	No. 8, No. 11,	No. 8, No. 11,	No. 8, No. 12,
Estimator		No. 12, No. 18	No. 12, No. 19	No. 12, No. 13	No. 19, No. 20
				No. 19	
Garman–Klass	No. 8, No. 12	No. 8, No. 11,	No. 8, No. 11,	No. 8, No. 12	No. 8, No. 11,
Estimator		No. 12	No. 12, No. 18		No. 12, No. 18,
					No. 19
Rogers–Satchell	No. 8, No. 12,	No. 8, No. 12,	No. 8, No. 12,	No. 8, No. 12,	No. 8, No. 11,
Estimator	No. 19	No. 19, No. 20	No. 19, No. 20	No. 19	No. 12, No. 16,
					No. 18, No. 19,
					No. 20

Based on symbol-sequence histograms. Notation as in Table 4.

**Table A2:** The sequences with zero frequency within the COVID-19 pandemic period.

	The Sequences with Zero Frequency Within the COVID-19 Pandemic Period				
	SPX	CAC	UKX	DAX	NKX
Parkinson	No. 8, No. 12,	No. 8, No. 12,	No. 8, No. 12,	No. 8, No. 12,	No. 8, No. 19
Estimator	No. 19	No. 18, No. 19	No. 18, No. 19	No. 13, No. 19	
Garman–Klass	No. 8, No. 12	No. 8, No. 12,	No. 8, No. 12,	No. 8, No. 12,	No. 8, No. 9,
Estimator		No. 16, No. 20	No. 20	No. 19	No. 11, No. 13,
					No. 15, No. 19
Rogers–Satchell	No. 8, No. 11,	No. 8, No. 11,	No. 8, No. 9,	No. 8, No. 9,	No. 8, No. 15,
Estimator	No. 12, No. 19	No. 12, No. 20	No. 10, No. 12	No. 11, No. 12,	No. 19, No. 20
				No. 19	

Based on symbol-sequence histograms. Notation as in Table 4.

Table A3 and Table A4 report the three most frequently observed sequences within the pre-COVID-19 and COVID-19 periods. These sequences are No. 1 (1,1,1), No. 4 (0,1,1), and No. 5 (1,1,2) (see Table 4). The main evidence is that the results are homogeneous since the most frequent sequences are exactly the same for all investigated volatility estimators and stock market indexes.

**Table A3:** The three most frequently observed sequences within the pre-COVID-19 period.

	The Three Most Frequently Observed Sequences Within the Pre-COVID-19 Period				
	SPX	CAC	UKX	DAX	NKX
Parkinson	No. 1 (467 times)	No. 1 (406 times)	No. 1 (407 times)	No. 1 (387 times)	No. 1 (430 times)
Estimator	No. 5 (50 times)	No. 4 (60 times)	No. 4 (62 times)	No. 4 (61 times)	No. 5 (53 times)
	No. 4 (39 times)	No. 5 (54 times)	No. 5 (57 times)	No. 5 (61 times)	No. 4 (51 times)
Garman–Klass	No. 1 (478 times)	No. 1 (365 times)	No. 1 (364 times)	No. 1 (360 times)	No. 1 (440 times)
Estimator	No. 5 (53 times)	No. 4 (60 times)	No. 4 (66 times)	No. 5 (64 times)	No. 5 (50 times)
	No. 4 (47 times)	No. 5 (51 times)	No. 5 (63 times)	No. 4 (59 times)	No. 4 (49 times)
Rogers–Satchell	No. 1 (483 times)	No. 1 (377 times)	No. 1 (399 times)	No. 1 (379 times)	No. 1 (458 times)
Estimator	No. 5 (45 times)	No. 4 (64 times)	No. 4 (59 times)	No. 4 (62 times)	No. 5 (49 times)
	No. 4 (34 times)	No. 5 (59 times)	No. 5 (50 times)	No. 5 (60 times)	No. 4 (45 times)

Based on symbol-sequence histograms. Notation as in Table 4.

**Table A4:** The three most frequently observed sequences within the COVID-19 pandemic period.

	The Three Most Frequently Observed Sequences Within the COVID-19 Pandemic Period				
	SPX	CAC	UKX	DAX	NKX
Parkinson	No. 1 (405 times)	No. 1 (454 times)	No. 1 (482 times)	No. 1 (426 times)	No. 1 (416 times)
Estimator	No. 5 (55 times)	No. 4 (55 times)	No. 5 (53 times)	No. 4 (60 times)	No. 5 (54 times)
	No. 4 (54 times)	No. 5 (49 times)	No. 4 (45 times)	No. 5 (54 times)	No. 4 (50 times)
Garman–Klass	No. 1 (412 times)	No. 1 (459 times)	No. 1 (426 times)	No. 1 (451 times)	No. 1 (483 times)
Estimator	No. 5 (49 times)	No. 5 (57 times)	No. 5 (60 times)	No. 5 (56 times)	No. 5 (46 times)
	No. 4 (46 times)	No. 4 (39 times)	No. 4 (51 times)	No. 4 (46 times)	No. 4 (38 times)
Rogers–Satchell	No. 1 (446 times)	No. 1 (506 times)	No. 1 (483 times)	No. 1 (473 times)	No. 1 (466 times)
Estimator	No. 5 (50 times)	No. 5 (47 times)	No. 4 (42 times)	No. 5 (53 times)	No. 4 (46 times)
	No. 4 (40 times)	No. 4 (43 times)	No. 5 (42 times)	No. 4 (39 times)	No. 5 (35 times)

Based on symbol-sequence histograms. Notation as in Table 4.
